# Race, ethnicity and COVID-19 vaccination: a qualitative study of UK healthcare staff

**DOI:** 10.1080/13557858.2021.1936464

**Published:** 2021-06-06

**Authors:** Charlotte Woodhead, Juliana Onwumere, Rebecca Rhead, Monalisa Bora-White, Zoe Chui, Naomi Clifford, Luke Connor, Cerisse Gunasinghe, Hannah Harwood, Paula Meriez, Ghazala Mir, Jessica Jones Nielsen, Anne Marie Rafferty, Nathan Stanley, Dorothy Peprah, Stephani L. Hatch

**Affiliations:** a Department of Psychological Medicine, Institute of Psychiatry, Psychology and Neuroscience, King’s College London, London, UK; b ESRC Centre for Society and Mental Health, King’s College London, London, UK; c Department of Psychology, Institute of Psychiatry, Psychology and Neuroscience, King’s College London, London, UK; d South London and Maudsley NHS Foundation Trust, London, UK; e Avon and Wiltshire Mental Health NHS Partnership Trust, Bristol, UK; f Nottinghamshire Healthcare NHS Foundation Trust, Nottingham, UK; g Cambridgeshire and Peterborough NHS Foundation Trust, Cambridge, UK; h Leeds Institute of Health Sciences, University of Leeds, Leeds, UK; i Department of Psychology, City University of London, London, UK; j Department of Adult Nursing, Florence Nightingale Faculty for Nursing, Midwifery and Palliative Care, King’s College London, London, UK; k London School of Hygiene and Tropical Medicine, London, UK

**Keywords:** Race, ethnicity, COVID-19, vaccine hesitancy, healthcare staff, discrimination

## Abstract

**Objective:**

COVID-19-related inequities experienced by racial and ethnic minority groups including healthcare professionals mirror wider health inequities, which risk being perpetuated by lower uptake of vaccination. We aim to better understand lower uptake among racial and ethnic minority staff groups to inform initiatives to enhance uptake.

**Design:**

Twenty-five semi-structured interviews were conducted (October 2020-January 2021) with UK-based healthcare staff. Data were inductively and thematically analysed.

**Results:**

Vaccine decision-making processes were underpinned by an overarching theme, ‘weighing up risks of harm against potential benefits to self and others’. Sub-themes included ‘fear of harm’, ‘moral/ethical objections’, ‘potential benefits to self and others’, ‘information and misinformation’, and ‘institutional or workplace pressure’. We identified ways in which these were weighted more heavily towards vaccine hesitancy for racial and ethnic minority staff groups influenced by perceptions about institutional and structural discrimination. This included suspicions and fear around institutional pressure to be vaccinated, racial injustices in vaccine development and testing, religious or ethical concerns, and legitimacy and accessibility of vaccine messaging and communication.

**Conclusions:**

Drawing on a critical race perspective, we conclude that acknowledging historical and contemporary abuses of power is essential to avoid perpetuating and aggravating mistrust by decontextualising hesitancy from the social processes affecting hesitancy, undermining efforts to increase vaccine uptake.

## Introduction

Addressing racial and ethnic discrimination is vital to global efforts in defeating COVID-19 (Bhala et al. 2020). Data from the UK, US and Europe find racial and ethnic minority groups have experienced disproportionately high rates of infection and mortality (Bhala et al. 2020; Office for National Statistics 2021; Nazroo and Becares 2020; Valeriani et al. 2020). Multiple and inter-related proximal causes of such inequalities have been suggested. For instance, Black, Asian and ethnic minority groups are more likely to experience chronic health conditions, associated with poorer COVID-19 related outcomes (Williamson et al. 2020), and are more likely to live in overcrowded households and accommodation with shared facilities or communal areas (Iacobucci 2020), in which COVID-19 is more prone to spread (Cevik et al. 2020). Some racial and ethnic minority groups are more likely to live in larger sized and multiple generation households for cultural, religious and/or affordability reasons, making challenges with social distancing unavoidable (Martin et al. 2020). They are also more likely to be employed as essential workers or less able to work from home, and therefore experience a greater risk of exposure at work or commuting (Office for National Statistics 2021). Underlying these more immediate risk factors is the cumulative impact of structural and institutional racism (Nazroo and Becares 2020).

### Vaccine hesitancy

Widespread global access and timely uptake of COVID-19 vaccination is key to halting the virus and minimising (or delaying) threat from new variants (Corey et al. 2020). However, refusal or delayed COVID-19 uptake is potentially a major hindrance in distribution (Harrison and Wu 2020) and may exacerbate existing health inequalities. Vaccine hesitancy is not specific to COVID-19, with differential uptake observed for other vaccines (e.g. seasonal influenza) which disadvantage racial and ethnic minority groups (Jain et al. 2017). Hesitancy is a ‘delay in acceptance or refusal of vaccination despite the availability of vaccination services’ (MacDonald 2015, 4163), occurring on a continuum from acceptance with no doubts to refusal with no doubts, with vaccine-hesitant individuals existing between these two stances. The World Health Organization’s (WHO) Vaccine Hesitancy Determinants Matrix (MacDonald 2015) acknowledges contemporary and historic contextual factors affecting hesitancy and that people may accept one vaccine while being hesitant about or declining another (Larson et al. 2014). However, it does not explicitly recognise or account for inequities in hesitancy which may inform current attempts to equitably enhance uptake.

For the COVID-19 vaccine, such inequities have been evident for racial and ethnic minority populations, for whom greater vaccine hesitancy has been found in the UK (Freeman et al. 2020; Robertson et al. 2021; SAGE 2021) and internationally (Robinson et al. 2020). There is concern that if acceptance is low among racial and ethnic minority communities, COVID-19 will widen existing inequalities (SAGE 2021).

### Vaccine hesitancy among healthcare staff

Addressing vaccine hesitancy and improving uptake among healthcare providers is a global public health issue (Karafillakis et al. 2016; Paterson et al. 2016) because healthcare workers are disproportionately exposed to infection (Evans et al. 2020). Further, the pressures of the pandemic and exposure to infection affect workplace absence and morale, in turn placing an undue burden on health service capacity (Abbasi 2021; Dacre 2021). Safety concerns; lack of vaccine knowledge or misinformation; lack of awareness about national guidelines; beliefs that one’s own vaccination does not benefit patients; concern about effectiveness and about necessity; societal endorsement; and workplace peer support are all reasons for vaccine hesitancy identified among healthcare staff cited internationally (Yaqub et al. 2014; Paterson et al. 2016).

Available US-based evidence about the influenza vaccine finds lower uptake and/or greater hesitancy among Black and Latinx healthcare staff groups (e.g. Lu et al. 2014). Racial and ethnic minorities are overrepresented within the UK healthcare workforce and may be expected to have lower institutional and vaccine trust given greater perceived discrimination and distrust within and outside of the health service (Rhead et al. 2020; Polling et al. 2020). They also disproportionately experience occupational exposure to (Pan et al. 2020) and deaths from COVID-19 amongst staff (Evans et al. 2020). However, there is little available evidence about mechanisms underpinning COVID-19 vaccine hesitancy among racial and ethnic minority healthcare staff to inform approaches to better support decision-making around vaccine uptake. This study therefore specifically addresses the following questions:

(1) How do healthcare staff make decisions about taking up a COVID-19 vaccine?
(2) How are these decision-making processes experienced by racial and ethnic minority healthcare staff groups, and how this is influenced by racism and discrimination?

## Materials and methods

Qualitative data were collected from two samples during phase two of the Tackling Inequalities and Discrimination Experiences in health Services (TIDES) study (www.tidesstudy.com), a UK-based project aiming to identify inequalities in health service use and exploring discrimination experienced by healthcare staff and service users (Rhead et al. 2020). Phase two was initiated in July 2020 to examine the national impact of COVID-19 on inequalities experienced by racial and ethnic minority health and social care staff groups. This was developed with and guided by a modified Delphi consensus process (Linstone and Turoff 1975) with an expert panel (advisory group) comprising clinical academics, health and social care staff and senior leaders, and a wider stakeholder opinion group of health and social care staff across England.

### Context

Data were collected between October 2020 and January 2021, when COVID-19 infection, hospitalisation and death rates rapidly rose twice across the UK, putting unprecedented strain on health and social care services. Data collection coincided with the rapid development and approval of initial COVID-19 vaccinations amidst widespread media coverage and discussion about the process of vaccine development, as well as government indications for how and when the vaccines would be rolled out. Data collection began just prior to roll-out and continued during the initial roll-out phases.

### Participants and sample selection

#### Healthcare staff (HCS) sample

Healthcare staff initially took part in the TIDES phase one survey (*n* = 931) detailed elsewhere (Rhead et al. 2020) and subsequent qualitative interviews (*n* = 46), including student nurses, healthcare assistants, and qualified nurses within the NHS recruited January 2019–February 2020. Staff agreeing to be recontacted (*n* = 45) were purposively sampled for phase two to include both racial and ethnic minority and white British staff across seniority levels. Criteria were based on evidence indicating differential exposure to discrimination by seniority and race/ethnicity and poorer career progression among racial and ethnic minority groups (NHS Equality and Diversity Council 2019). The current study includes TIDES phase two data.

#### Senior management sample

We combined snowball and key informant sampling (Marshall 1996) for TIDES phase two. Our targeted sampling approach included: high-level management from national health bodies, senior-level clinicians with management responsibilities; and non-clinical senior-level staff in equality, diversity & inclusion (EDI)-related roles. Advisory and stakeholder opinion groups helped identify potential key informants meeting these criteria. Of those, we prioritised recruitment to incorporate both racial and ethnic minority and White British participants nationally. Senior sample recruitment was continued alongside iterative data analysis until saturation.

#### Recruitment

Recruitment and data collection occurred between October 2020 and January 2021. Potential participants received an e-mail invitation. Those expressing interest were sent an information sheet with details about data confidentiality and study withdrawal. Three maximum further contact attempts were made. Participants recruited were assigned a unique ID number to complete a digital consent form prior to interview. Participants provided socio-demographic information (gender, race and/or ethnicity, migration status, religion) and were offered £15 shopping vouchers in appreciation for their time.

#### Data collection

Online semi-structured interviews (45–60 minutes) were recorded with consent. Topic guides were developed and refined with advisory and stakeholder opinion groups. Interview domains covered racial and ethnic inequalities experienced by staff (e.g. witnessing and experiencing workplace discrimination, bullying and harassment). Current analyses focus on data pertaining to questions and probes about COVID-19 vaccination (e.g. beliefs about uptake and implementation; vaccine and roll-out concerns).

Healthcare staff interviews were conducted by experienced TIDES qualitative researchers. External EDI experts with experience in interviewing NHS managers conducted senior management interviews. Data were transcribed verbatim, removing identifying information.

#### Ethical approval

Ethical approval was granted by the King’s College London Research Ethics Committee for Psychiatry, Nursing and Midwifery (HR-17/18-4629; RESCM-19/20-4629; RESCM-20/21-4629) and NHS Health Research Authority (18/HRA/0368).

#### Analysis

Data were analysed thematically using an inductive approach (Braun and Clarke 2012) by a racially, ethnically and gender diverse research team. Following familiarisation, one researcher descriptively coded transcripts, using these to develop an initial coding framework. Two other researchers descriptively coded a subset of the transcripts, together discussing and refining the coding framework, applying it to all transcripts after inputting into NVivo (QSR International Pty Ltd 2018) to support data management. No major interpretative differences were raised, but nuances were developed through discussion. Codes were grouped into themes reflecting key patterns in the data relevant to the research questions, continually checking and refining against the raw data, actively looking for similarities and differences both within and across datasets, and looking for patterns by participant characteristics. Themes were defined, described and labelled. Patterns between themes and our narrative interpretations were discussed within the research team and with advisory and stakeholder opinion groups.

## Results

### Sample

The analytic sample included 17 healthcare staff and eight senior management members. Of 25 participants, nearly three-quarters identified as female, two-thirds identified as a racial or ethnic minority group, over a third were born outside the UK and three-quarters specified a religious faith. Qualified nurses and senior clinicians each comprised two-fifths of the sample, the remainder were students, healthcare assistants or EDI specialists. Around two-thirds of interviews occurred just before, and a third just after roll-out of COVID-19 vaccinations in the UK (Table 1).

### Vaccine stance: accepters, decliners and hesitants

Participants’ vaccine stances lay on a continuum from those who would immediately accept if offered or had already been vaccinated (herein referred to as ‘vaccine accepters’), to those who were certain that they would decline (‘vaccine decliners’). Between these stances were people defined as ‘vaccine hesitant’. Hesitancy reflected a nuanced stance, from those who reported feeling undecided to those likely to accept eventually but wanted to wait and see before deciding. Just over half of participants were vaccine accepters, over a third expressed hesitant views, while a minority reported that they would decline.

I wouldn’t be the first one to go for the vaccine, but I might be the 10th or the 11th, or the 100th if that makes sense. (Hesitant, Asian, senior clinician, before roll-out)

We also asked participants whether they thought any staff groups would be (or were experienced as) more or less likely to take up the vaccine. Among those identified were staff who would normally decline the influenza (‘flu) vaccine (e.g. due to underlying health conditions or other potential risk factors like pregnancy); and that racial and ethnic minority groups may be (or were, following roll-out) particularly hesitant (or ‘suspicious’) about vaccine uptake. To help untangle the underlying causes of hesitancy and suspicion, we describe the decision-making process within one overarching theme, ‘weighing up risks and benefits for self and others’.

### Weighing up risks and benefits for self and others

For those hesitant about the vaccine, decision-making was described as an active appraisal process, a balancing act and personal decision requiring calculation of potential risk from the vaccine and from COVID-19, against the potential benefits of being vaccinated. Importantly, this stance was not necessarily static.

I was neutral for a long time on it and only last week, I made a decision to have the vaccine and I’ve had it and um[...] I weighed up the evidence and come to that decision for myself. But as I say, it is a personal thing that people have to decide for themselves. (Accepter, Black Caribbean, senior management, after roll-out)

Some immediate accepters also referred to this weighing up process but the balance more clearly favoured vaccination. Parameters influencing these calculations were represented by the sub-themes ‘fear of harm’, ‘moral or ethical objections’, ‘potential benefits to self and others’, ‘information and misinformation’, and ‘institutional or workplace influence’. Comparing vaccine stances, decliners focussed on perceived risk (fear of harm to self) and less on benefits to self and others. In contrast, hesitants described both fears of harm (particularly unknown long-term effects) as well as benefits to self and others, equally. Accepters also described fears but primarily in relation to what they had heard other’s say; instead, they most commonly focussed on vaccination benefits.

### Fear of harm

This theme referred to fears about harm, influenced by perceived trust in the vaccine, personal vulnerability and risk, as well as precedence and past experience. Harm included immediate side effects such as adverse health impacts for people with underlying conditions (whether known or to be revealed only after being vaccinated), developing COVID-19 symptoms and, particularly, fears of longer-term effects. Such effects were often unspecified but included, for example, birth defects or effects on fertility.

### Trust in the vaccine

Fears of harm were grounded primarily in reservations about the speed of vaccine production, trialling and implementation. Participants had strong perceptions that fast-tracking may have led to corner-cutting, lack of robust testing and inaccurate assessments about the risk of side effects and/or effectiveness against COVID-19. Allied to these fears was a perceived lack of sufficient research or clinical evidence. Specifically, concerns about longer-term side effects were underpinned by perceived lack of longitudinal data on its effects. Such doubts were mentioned by the majority of hesitants, all decliners but none of the accepters.

I wouldn’t take it up if offered, um. I’m quite dubious of taking a vaccine that’s only been trialled for about 9, I don’t even know, 9 months? And I think medications need to look at not only the short-term effects but the longitudinal effects of taking a vaccine medication. Um as a young woman of fertile age I don’t want anything that could potentially either harm any fertility maybe in the future. (Decliner, Black African, HCS, before roll-out)

A vaccination that should have taken 10–20 years to develop and test properly has been produced in a matter of months. And, um ... so it hasn’t been -it hasn’t been tested and it hasn’t been scrutinised the way it should have been. (Hesitant, Black Caribbean, HCS, after roll-out)

While some accepters acknowledged concerns about fast-tracking, in contrast, some expressed particular confidence in the development process.

I’m fine with it, like I mean it’s been approved in this country, it’s been approved in other countries now as well, like. I mean I have the flu vaccine every year and like we’ve had patients who have bought into the anti-vaxx stuff, but. You know, we’re medical professionals so we shouldn’t be getting drawn into that. (Accepter, White British, HCS, before roll-out)

Among certain participants, vaccine mistrust reflected mistrust in government and pharmaceutical companies. For some racial and ethnic minority staff, particularly Black African and Black Caribbean and other Black groups, suspicion and mistrust in the vaccine development process was particularly salient, grounded in fears and recollections of experiences related to poor and unethical research practise.

BME staff have been less likely to opt into receiving the vaccine [...] I think there’s something around how … If you look at things historically, Black and Asian communities have been misused in research [...] we have been abused and violated in previous vaccination trials and we can’t deny that. (Decliner, Asian, senior management, after roll-out)

Trust also related to beliefs about vaccine effectiveness and uncertainty surrounding protection against transmission. Some participants questioned whether there was sufficient evidence on vaccine efficacy to warrant the immediate and personal risk of being vaccinated; which coincided with a lack of clarity about the available information. For instance, media reporting headline figures about varying vaccine efficacy was a source of considerable uncertainty.

I know this coronavirus is gonna mutate into another virus at some point anyway, so you know, if it’s going to be 21, coronavirus 21, and. If you’ve been vaccinated this time around will it fight the coronavirus um will it fight coronavirus 21? (Hesitant, White British, HCS, before roll-out)

I haven’t really seen a lot of, information on it yet or done a lot of research on it just kind of what I’ve seen on TV and stuff, um but I mean. I guess it can only be a good thing if it stops it from getting worse in the future. I know it’s only about 90% accurate, whatever. (Accepter, Asian, HCS, before roll-out)

Uncertainty also highlighted a margin of error into which some participants worried that they might personally fall.

### Personal vulnerability and risk

For some, worries about adverse effects of the vaccine tended to be linked to specific pre-existing vulnerabilities. In participants classified as decliners, this reflected unease about known underlying health conditions also affecting uptake of other vaccines.

So when the vaccine coming now, I say no I will not- I need to read, I need to know 90% for me you know it, it could be that you know especially my system’s rubbish, I have vitamin D deficiency, I have anaemia now, I don’t know why. So you know my body has, I don’t know whether to fight the new thing. (Decliner, Other ethnic group, HCS, before roll-out)

Among hesitants, there were common concerns that unknown personal vulnerabilities may arise leading them to experience harm from vaccination.

I spoke to my wife about it and because we have a 5-year old son. She’s like to me, one of us should have it and one of us should not have it because then we’re covering both, you know, if anything happens - and I grow an extra limb [laughs], I’m exaggerating again. I don’t know, something might happen within my respiratory - I might be more prone to respiratory illness or whatever it might be. (Hesitant, White British, HCS, before roll-out)

### Precedence and past experience

Other grounds for apprehension were related to prior experiences of vaccines or treatment which had – only after widespread roll-out – been found to cause more widespread harm (e.g. Thalidomide).

I don’t know if it would but you know like, I guess it’s different but like [drug name] for our patients like they only just found out that that has birth defects and can cause disabilities. (Decliner, Black African, HCS, before roll-out)

Both hesitants and decliners raised negative past vaccine experience as a source of doubt. In contrast, accepters all referred to past experience in a normalising way – comparing the COVID-19 vaccine to the ‘flu or other routine vaccines for healthcare staff.

When I joined the Trust I had to go through occupational health and have a whole load of vaccines. So I feel like it’s just going to be another one added in. (Accepter, White British, HCS, before roll-out)

It was frequently mentioned that COVID-19 vaccine hesitancy or decline is not necessarily unexpected because it also commonly occurs with the ‘flu vaccine, which is routinely offered to healthcare staff.

A lot of people don’t have the flu vaccine for example, and not putting race against the flu vaccine but I’m just saying that a lot of people, whatever race they are, they don’t take the flu vaccine, and there will be this COVID vaccine where people won’t take the COVID vaccine. (Hesitant, Asian, HCS, before roll-out)

### Religious or ethical objections

The second theme influencing the decision-making process was ‘religious or ethical objections’. This was less commonly endorsed by participants and tended to be cited about other people’s stance rather than their own.

A lot of people um use kind of religious reasons for not taking it. Um ‘cause there was something in the news today about some of the components of the vaccine is um, genes from kind of unborn babies or something crazy like that so, a lot of religious people will not take the vaccines for those sorts of reasons. (Accepter, Asian, HCS, before roll-out)

Some staff just are hesitant, and you would say that some of our BAME staff particularly are very hesitant – even, and in the matter of fact, the ones who are even high risk really very high risk, shielding, even they - some of them are still reluctant. That comes out quite strange, but you know it might be something to do with cultural beliefs. (Accepter, Asian, senior management, after roll-out)

We explored variation by reported religiosity; hesitants and accepters were fairly evenly split across those who did or did not identify themselves as being religious though decliners did identify as religious. However, there were no clear patterns when examining reasons for hesitancy or refusal and decliners did not cite religious or ethical objections as affecting personal decision-making.

### Potential benefits to self and others

Accepters and hesitants tended to focus more on the potential benefits of vaccination to themselves and others. This included protecting yourself from the virus, protecting other people by reducing the likelihood of transmission, and more broadly supporting the effort to halt disease spread. Vaccine development and uptake were frequently positioned as key to facilitating and expediting a return to pre-COVID-19 life. While accepters included participants from across seniority levels, senior nursing and management staff particularly emphasised the importance of protecting others.

### Protecting yourself and others

While protecting oneself from contracting COVID-19 was discussed, there was also a corresponding sense of moral or social responsibility (a ‘duty of care’) – protecting yourself would also protect others. This motivation reflects concerns about bringing infection home or to vulnerable people outside work, as well as patients. There was acknowledgment and shared endorsement of others’ concerns about potential risks; however, some also expressed explicit disapproval or judgement for vaccine decliners, labelling such a decision as ‘selfish’ for prioritising personal preference or concerns.

It’s not all about me, I may be a healthy person but obviously all the vulnerable people, so we might be carrying something that is deadly for them. Who knows, it might be deadly for my kids. Me protecting myself is not just about protecting myself, it’s about whoever surrounds me as well. (Accepter, White Other, HCS, before roll-out)

Direct exposure to the impact of COVID-19 while treating patients, and reflected in intensified workplace pressures since the outbreak, contributed to a sense of urgency. This was a strong counterbalance against fears about potential harm.

I can imagine the majority of staff will take it, especially in the healthcare setting because you see a lot of it first-hand how it’s affecting people so I can’t imagine a lot of people would say no to it. (Accepter, Asian, HCS, before roll-out)

From the worker perspective I suppose there is this um, obligation in many respects to have the vaccine to make sure that you are reducing the risk of either contracting COVID yourself or spreading COVID in the health service, given the pressures that we’re under. (Hesitant, Asian, HCS, before roll-out)

Hesitants also discussed potential benefits related to the protection of self and others and a similar moral stance. However, more so than for accepters, this tended to be caveated as dependent upon reports of vaccine effectiveness. Some participants also cited uncertainty about the necessity of vaccination (e.g. being currently fit and healthy or having already had the infection). There was some indication that staff not working in direct patient care may also need more convincing of the personal benefits of vaccination.

I think I would probably get it, just because … I’ve had it [COVID-19] before I know there’s some kind of like, resistance to the antibodies at the beginning but then they say that the antibodies decrease and you’re not really protected anymore from a second infection. So I know that I would be at risk again and that I should probably have it. But it is just kind of weighing up like, am I likely to get it again or what could be the long-term effects of these vaccines I don’t know, but I’m still undecided. (Hesitant, White British, HCS, before roll-out)

### Getting back to normality

Another key rationale for accepting vaccination was to get back to normality. This was endorsed mainly by accepters and only one hesitant, who wanted to travel to see family. They felt that this involved a kind of social contract, relying on other people also being vaccinated for it to work.

We have to go back to normal. We have to start visiting our families again, and it’s for our patients as well. And then you need like, for it to work you need a percentage of the population to be getting the vaccine, otherwise it won’t. It won’t work. (Accepter, White Other, HCS, before roll-out)

### Information and misinformation

Fears of harm, beliefs about benefits to self and others and, to some extent, religious or ethical objections, were all affected by uncertainty. This arose from suspicions about fast-tracking and a perceived lack of research evidence as described above, but also from the nature and sources of information available about the vaccine, which many felt lacked clarity. For hesitants and decliners, in particular, a lack of trustworthy information left a void into which doubt could be fuelled by media speculation and misinformation. There was mistrust that information could be manipulated to fit government or institutional agendas, alongside confusion about messaging.

I guess if the government agenda is for the vaccine to be rolled [out], definitely there are going to be manipulation within the news. (Hesitant, White Other, HCS, before roll-out)

This was a key area in which racial and/or ethnic minority staff, particularly those experiencing language barriers, may be affected. Trust in vaccine information is affected by historical abuses as alluded to earlier but also by how relatable and accessible that information is and it’s source.

It’s understanding people’s cultures and being culturally competent, culturally aware. And having, you know, people speaking to their own communities. Um people who are in positions like myself speaking to your own communities and family members to give out positive messages to the family members and wider community to say actually, I would take the COVID vaccine and therefore you should be doing it as well. Um and these are the benefits and these are the risks. But if you’re going to get somebody else who doesn’t look like you, doesn’t sound like you, doesn’t speak your language; that trust is not there, necessarily. (Hesitant, Asian, HCS, before roll-out)

Many participants, particularly accepters and hesitants, were careful to distance themselves from ‘anti-vaxxers’, referring to those who reject *all* vaccinations for reasons including conspiracy theories. For some, this came with a sense of social desirability about being vaccinated – highlighting that they were usually pro-vaccine, first in line to get the ‘flu jab or would encourage others to be vaccinated if they were prevented by health reasons.

I’m still holding the point that I’m always against all these anti-vaxxers saying oh this is just a, you know, something that er vaccine companies or pharmaceutical companies have created just to get some more money. (Accepter, White Other, HCS, before roll-out)

### Institutional or workplace influence

Institutional or workplace influence could vary in intensity from making vaccination obligatory, putting ‘pressure’ on, or ‘encouraging’ staff or specific groups to accept. Hesitants and decliners discussed institutional influence very differently to accepters. The former – talking mainly before or just at the start of vaccine roll-out – expressed worries about being ‘forced’ by employers to be vaccinated. This was reinforced by media speculation and workplace rumours about whether there would be sanctions from employers (e.g. job loss, redeployment from patient-facing roles) or government (e.g. travel restrictions) if they declined. Mandating and being ‘forced’ was a worry because it would restrict autonomy in ‘weighing up’ their vaccination decision. Others pointed to the unfairness about being forced to take something with a perceived limited evidence-base.

I don’t think it’s fair to force people to take something that’s only been trialled and tested for six months. (Decliner, Black African, HCS, before roll-out)

Accepters discussed mandating the vaccine differently. With the exception of one senior clinician who strongly supported mandating the vaccine, others in this group (mainly in the healthcare staff sample) were ambivalent or expressed resignation about the prospect – expecting not to have a choice or to experience pressure.

I think I’d be kind of crazy not to, I’d almost feel like they’re going to make it mandatory anyway, I’d imagine they would not make it an option to take it. (Accepter, Asian, HCS, before roll-out)

Such resignation was not mirrored by senior management who (talking mainly after the onset of vaccination) emphasised the importance of personal choice and anticipated push-back to mandatory vaccination.

People are making an informed choice um- but I don’t think we can mandatory- I think there’d be- I think there’d be um- civil war if we’d mandated that people get jabbed with this virus-with the vaccine. (Accepter, White British, senior management, after roll-out)

Nonetheless, participants from all vaccine stances expected to receive direct and indirect forms of ‘pressure’ or proactive ‘encouragement’ from their organisation to be vaccinated. Several participants anticipated racial and ethnic minority staff groups would be or feel particularly pressured (or encouraged) to take it up.

Because people from um BME backgrounds have been identified as higher risk they could potentially also be more pressured into getting it, of getting the vaccine? And perhaps they um, they wouldn’t want it for their own personal reasons, but then my worry and also I think a lot of people’s worry at the moment is what is the NHS going to say in terms of getting the vaccine; is it going to mandatory, are people going to be threatened with, you know, job loss or that kind of thing if they don’t want to get it. (Hesitant, White British, HCS, before roll-out)

There was an expressed sense from some participants that putting *pressure* on racial and ethnic minority staff groups, however well intended, was likely to fuel suspicion and resistance.

Someone said, ‘oh you know they going to force us to have it’, I go ‘no no I’m not going to have any vaccine’, you know when they talking they say oh they are going to force us- I said ‘no one can force us to have anything’. (Decliner, Other ethnic group, HCS, before roll-out)

Alternatively, approaches which *encouraged* uptake (through targeting messages, providing information directly addressing specific concerns raised by racial and ethnic minority staff, and leveraging social norms) were expected to yield greater success.

We’ve got a number of our senior staff that have had the jab that are – that we’re using in promotions to say you know, like doctors, consultants and um other sort of more sort of more senior nurses that are BAME to say, ‘I’ve had my jab done, get yours done’. (Accepter, White British, senior management, after roll-out)

Moreover, not involving affected staff in efforts to promote uptake may limit the effectiveness of campaigns.

I’m not sure if they’ve done any particular work with the BME staff network to understand why people might be apprehensive about the vaccine. There’s not been any direct kind of involvement, or them reaching out to understand what the issue is. (Decliner, Asian, senior management, after roll-out)

## Discussion

We qualitatively explored views about COVID-19 vaccination among healthcare staff to better understand influences on decision-making, particularly among racial and ethnic minority staff groups. A single overarching theme explained vaccine decision-making as a process of weighing up potential risk and benefits for themselves and for others. Five sub-themes reflected key influential parameters: fear of harm; religious and/or moral objections; potential benefits to self and others; information and misinformation; and, institutional or workplace pressure. While aspects of each theme overlapped with concerns reported in general population studies (e.g. fears about side effects and fast-tracking, beliefs in vaccine effectiveness), participants also highlighted influences specific to healthcare staff (e.g. duty of care, institutional/workplace pressure, exposure to the impact of infection, experience with other routinely offered vaccines at work). Some of these were particularly pertinent for racial and ethnic minority group staff, who also experienced additional inter-related grounds for vaccine hesitancy.

This included suspicions and fear around being pressured to be vaccinated, racial injustices in vaccine development and testing, religious or ethical concerns, and legitimacy and accessibility of vaccine messaging and communication. Except religious/moral objections (only reported indirectly), all these factors reflected greater mistrust in institutions involved in vaccine promotion. We take a critical race theory perspective (Crenshaw et al. 1995), discussing how racism and racialised stratification processes underpin observed and anticipated inequities in vaccine hesitancy; result in influences affecting hesitancy common to the wider population being weighted more heavily for racial and ethnic minority groups; as well as provoking additional grounds for hesitancy. Furthermore, we consider how discriminatory social processes also influence the efficacy of interventions designed to enhance vaccine uptake.

### Comparisons with previous literature

Limited available evidence suggests lower vaccine uptake among Black healthcare staff is due to higher levels of vaccine concern (Ojha et al. 2015). While aligned with our findings and helping identify putative targets for enhancing uptake, such concerns were reported by healthcare staff across racial and ethnic groups in our study and mirror mechanisms underpinning vaccine hesitancy generally (Freeman et al. 2020; Larson et al. 2014; Peprah et al. 2016). Moreover, it leaves the question of *why* they hold these concerns to a greater extent. In response, we draw on aspects of the ‘Vaccine Hesitancy Determinants Matrix’ (MacDonald 2015) which were raised by participants (Table 2).

### Contextual influences

Our findings illustrate the salience of contextual influences on vaccine hesitancy among racial and ethnic minority groups. This is supported by research among US African diaspora communities, identifying issues such as government and pharmaceutical company mistrust, prior racial injustices linked to unethical research, social media misinformation, and concerns about vaccine prioritisation being experimentation (Ateghang-Awankem and Anchang 2020). UK-based research indicates trust in COVID-19 information received from government, scientists and health-related institutions is lower among Black, Asian and minority ethnic groups (Wellcome Monitor 2020) and mistrust is associated with COVID-19 vaccine refusal or hesitancy (Murphy et al. 2021).

Mistrust is grounded in wider inequalities beyond COVID-19; racial and ethnic minority groups are more likely to experience socio-economic disadvantage, exposure to and anticipation of discrimination throughout their lives and across generations (e.g. Rhead et al. 2020; NHS Equality and Diversity Council 2019; Williams and Cooper 2020). Since the outbreak, intensified awareness of disproportionate infection and death rates likely serves as a constant reminder of the structural and institutional racism underpinning these inequalities.

The legacy of medical experimentation and unethical research, including neglect and lack of accountability for adverse effects, on racially minoritised communities, further exacerbates mistrust in Western pharmaceutical companies and vaccines (Washington 2006; Jegede 2007; Scharff et al. 2010; Centres for Disease Control and Prevention 2020). This includes publicised suggestions about the experimentation of COVID-19 vaccines in Africa (BBC News 2020).

We found messaging and communication also influenced vaccine hesitancy. Public health messaging not tailored or relatable to different racial and/or ethnic communities may add, or fail to address uncertainty, confusion and vaccine mistrust, and reduce information accessibility (Ojha et al. 2015). In the UK, White Other, Asian and particularly, Black and mixed racial/ethnic groups are less likely to report finding information about COVID-19 clear compared to White British respondents (Wellcome Monitor 2020). Our findings illustrate that this does not reflect ‘lack of understanding’ but perceived legitimacy of the communication and (for some) language barriers, reinforcing the importance of communication from members of their own communities (SAGE 2021). Media misinformation was reported to exacerbate this, particularly impacting faith communities’ concerns about vaccine constituents (Sherwood 2021).

### Individual and group influences

As supported by our findings, social and peer influences include prior experience of knowledge and awareness about, and perceived risks and benefits of vaccination. Previously identified misconceptions among US-based African diaspora, such as the vaccine weakening their already strong enough immune systems to combat infection, further exemplify the need for nuanced uptake initiatives (Ateghang-Awankem and Anchang 2020). Further, trust in the health system and vaccine providers influences the extent to which immunisation is a social norm within communities, perceived as unnecessary or even harmful (MacDonald 2015). Lack of inclusion of racial and ethnic minority groups in research (Treweek et al. 2020), also likely raises concerns about the applicability of effectiveness and safety evidence.

### Vaccine or vaccination-specific influences

Reasons for hesitancy related specifically to vaccines or the vaccination process mainly pertained to suspicions about fast-tracking, pressure to be vaccinated, and perceived legitimacy of the evidence-based. Racial and ethnic minority staff groups may be more influenced by these factors due to the above issues, as well as prior adverse experiences with premature vaccine distribution (e.g. Sanofi’s dengue vaccine) (Dyer 2019).

### Strengths and limitations

Limited research exists examining vaccine hesitancy specifically among racial and ethnic minority groups, resulting in little evidence on hesitancy reduction interventions to draw on (SAGE 2021). We examined COVID-19 vaccination perceptions using timely data collected just prior to and after the UK vaccine roll-out. Nested within an ongoing project examining the impact of COVID-19 on inequalities experienced by racial and ethnic minority healthcare staff, our research was well placed to examine vaccine hesitancy and lower uptake. Although a key strength, the immediacy and severity of the issue may have influenced how participants framed their responses. It is possible that social desirability influenced senior management interviews conducted at vaccine roll-out outset – the need to set an example, and/or alignment with institutions promoting uptake. Interviews before roll-out may have reflected wider uncertainty about operational processes. As with other qualitative studies, we cannot claim generalisability; however, findings do support and refine existing theory and evidence underpinning vaccine hesitancy. Our study was developed with healthcare staff and interpretations have been refined through discussion among a racially and ethnically inclusive team of researchers and healthcare practitioners.

### Implications

Findings suggest that existing vaccine hesitancy models may need to be developed further in relation to racial and ethnic minority and religious communities for whom recognised influences are shaped by discriminatory social and institutional processes affecting mistrust. Findings also emphasise the importance of considering intersections between race/ethnicity, language, age, gender and parenthood. For enhancing uptake, we suggest no single approach will encourage uptake among racial and ethnic minority communities and healthcare staff, and support recommendations focussing on increasing trust and influencing social norms (UK Scientific Advisory Group for Emergencies (SAGE) 2021) and taking into consideration prior evidence on adapting public health interventions to racial and ethnic minority populations (Davidson et al. 2013). This involves collaborating with community-based organisations to develop tailored messaging and vaccine delivery through trustworthy and legitimate channels; targeted educational campaigns addressing specific vaccine concerns; and increased accessibility through community and workplace-based delivery. Our results indicate that meaningful and respectful community engagement and acknowledgment of historical and contemporary abuses of power is key.

For healthcare staff, our findings indicate that approaches to encourage uptake (e.g. promotional materials, engaging racial and ethnic minority staff groups to address specific concerns, and in-house vaccinations) may help increase trust through peer social norms and greater convenience. However, they are unlikely to be sufficient without acknowledging, validating and actively counteracting deep concerns linked to past and ongoing discrimination. Our study also indicates the centrality of personal decision-making; discriminating against hesitant staff will likely further alienate and intensify mistrust, undermining attempts to increase uptake.

### Conclusions

Lower COVID-19 vaccine uptake among racial and ethnic minority groups risks exacer-bating health and social inequalities. Concerns tipping the balance towards vaccine hesitancy are weighted more heavily for these groups for reasons broader than the vaccination itself. Instead of generalised approaches to encouraging uptake, they should be tailored to the nuanced concerns within and between different groups; transparent in acknowledging the root causes of concerns; and considerate of intersectional social statuses. Importantly, approaches must avoid perpetuating and aggravating mistrust by decontexualising hesitancy from underpinning social processes and not disproportionately pressuring, discriminating, or shaming marginalised communities for being hesitant. Community-led and engaged approaches are key though must be supported by commitments to address fundamental causes of COVID-19 inequalities rather than laying responsibility on those most affected.

## Figures and Tables

**Table 1:** Sample characteristics.

	*n*	%
**Gender**		
Male	6	24
Female	18	72
Other	1	4
**Race/ethnicity**		
Asian	4	16
Black African	2	8
Black Caribbean	4	16
White British	11	44
White Other	3	12
Other	1	4
**Migration status**		
Migrant	9	36
Non-migrant	16	64
**Religion**		
Religion specified	18	72
Specified no religion	6	24
**Sample**		
Healthcare staff	17	68
Senior management	8	32
**Timing**		
Before UK vaccine roll-out	16	64
After UK vaccine roll-out	9	36

**Table 2:** World Health Organization’s Vaccine Hesitancy Determinants Matrix (reproduced from MacDonald 2015, 4163).

**Contextual influences** Influences arising due to historic, socio-cultural, environmental, health system/institutional, economic or political factors	(a) Communication and media environment (b) Influential leaders, immunisation programme gatekeepers and anti- or pro-vaccination lobbies (c) Historical influences (d) Religion/culture/gender/socio-economic (e) Politics/policies (f) Geographic barriers (g) Perception of the pharmaceutical industry
**Individual and group influences** Influences arising from personal perception of the vaccine or influences of the social/peer environment	(a) Personal, family and/or community members’ experience with vaccination, including pain (b) Beliefs, attitudes about health and prevention (c) Knowledge/awareness (d) Health system and providers – trust and personal experience (e) Risk/benefit (perceived, heuristic) (f) Immunisation as a social norm vs. not needed/harmful
**Vaccine/vaccination – specific issues** Directly related to vaccine or vaccination	(a) Risk/benefit (epidemiological and scientific evidence) (b) Introduction of a new vaccine or new formulation or a new recommendation for an existing vaccine (c) Mode of administration (d) Design of vaccination programme/Mode of delivery (e.g. routine programme or mass vaccination campaign) (e) Reliability and/or source of supply of vaccine and/or vaccination equipment (f) Vaccination schedule (g) Costs (h) The strength of the recommendation and/or knowledge base and/or attitude of healthcare professionals
